# Viral Determinants of Virulence in Tick-Borne Flaviviruses

**DOI:** 10.3390/v10060329

**Published:** 2018-06-16

**Authors:** Eliza M. Kellman, Danielle K. Offerdahl, Wessam Melik, Marshall E. Bloom

**Affiliations:** 1 Laboratory of Virology, Rocky Mountain Laboratories, NIAID, NIH, Hamilton, MT 59840, USA; ekellman@usc.edu (E.M.K.); offerdahld@niaid.nih.gov (D.K.O.); 2 School of Medical Sciences, Orebro University, SE-703 62 Örebro, Sweden; wessam.melik@oru.se

**Keywords:** tick-borne encephalitis virus, tick-borne flavivirus, virulence, neuropathogenesis

## Abstract

Tick-borne flaviviruses have a global distribution and cause significant human disease, including encephalitis and hemorrhagic fever, and often result in neurologic sequelae. There are two distinct properties that determine the neuropathogenesis of a virus. The ability to invade the central nervous system (CNS) is referred to as the neuroinvasiveness of the agent, while the ability to infect and damage cells within the CNS is referred to as its neurovirulence. Examination of laboratory variants, cDNA clones, natural isolates with varying pathogenicity, and virally encoded immune evasion strategies have contributed extensively to our understanding of these properties. Here we will review the major viral determinants of virulence that contribute to pathogenesis and influence both neuroinvasiveness and neurovirulence properties of tick-borne flaviviruses, focusing particularly on the envelope protein (E), nonstructural protein 5 (NS5), and the 3′ untranslated region (UTR).

## 1. Introduction

Tick-borne encephalitis virus (TBEV) is the most medically relevant tick-borne flavivirus (TBFV), which together with Powassan/deer tick virus (POWV), Omsk hemorrhagic fever virus (OHFV), Kyasanur Forest disease virus (KFDV), louping ill virus (LIV), and the non-pathogenic Langat virus (LGTV) form the TBEV serocomplex. The TBEV serocomplex is part of the *Flavivirus* genus (family *Flaviviridae*), which includes the more well-known mosquito-borne flaviviruses (MBFV): West Nile virus (WNV), dengue virus (DENV), Japanese encephalitis virus (JEV), yellow fever (YFV), and Zika virus. The TBFVs generally circulate in Europe and Asia and are vectored by *Ixodidae* ticks, although alimentary transmission has also been documented [1]. TBEV, itself, is further divided by geographical location into three subtypes, European (TBEV-Eu), Siberian (TBEV-Si), and far-eastern (TBEV-FE). Although there are currently two European, two Russian, and one Chinese vaccines available, TBEV is estimated to cause 10,000 to 15,000 cases per year worldwide, and the incidence has increased almost 400% in the last 30 years [2,3,4,5]. POWV is the only recognized TBFV in North America. Infection with TBFVs is often asymptomatic, however more severe cases can develop and follow a biphasic course. Following an incubation period of 5–14 days, the first phase is generally characterized by fever, headache, muscle pain, and malaise. After the first phase resolves, some patients will develop the second phase of disease, which involves meningitis, meningoencephalitis, and meningoencephalomyelitis [6]. Up to 40% of these cases result in long-term neurological sequelae following encephalitis [7].

Neuropathogenesis of flaviviruses consists of two distinct properties of the virus: neuroinvasiveness and neurovirulence. Neuroinvasiveness relates to the virus’ ability to invade the CNS after virus transmission, while neurovirulence relates to the virus’s ability to enter cells in the CNS and establish a productive infection [8]. Following inoculation by tick bite, initial infection, and replication is thought to occur in dendritic cells in the subcutaneous tissues, which then transport the virus to draining lymph nodes. Replication in the lymph nodes leads to viremia and the establishment of a systemic infection [4,9]. How the virus crosses the blood–brain barrier (BBB) and gains access to the central nervous system (CNS) remains largely unknown, although it is unlikely to involve breakdown of the BBB, as virus replication is detectable in the CNS prior to BBB disruption [10]. Rather, a recent study by Palus et al. suggested a role for direct infection of the BBB endothelial cells in viral entrance into the CNS [11]. Regardless, the virus’ ability to induce high levels of viremia appears to be directly correlated to its ability to invade the CNS, or its neuroinvasiveness [12].

Once in the CNS, the virus primarily targets neurons, although other cell types may be infected as well [13,14]. In vitro studies of immortalized cell lines have shown evidence for direct virus-induced damage to neurons and cytopathic effects [15,16]. However, other studies have shown persistent infection of primary neurons, and clinical studies have not found significant evidence for direct neuronal damage, such as cytopathic effect or activation of apoptotic cascades [17,18]. Rather, immunological factors seem to contribute more to tissue damage following CNS invasion. Recently, CD8+ T cells and B cells were found to have a pathological impact on disease progression in a TBEV animal model, whereas CD4+ T cells had a protective effect [19]. This does not, however, entirely rule out direct viral-induced damage, whether by apoptosis or necrosis. In addition, TBEV infection has been shown to inhibit neurite outgrowth and disrupt host mRNA transport along dendrites [20,21,22]. Thus, neuronal damage and immunopathological effects appear to be mainly responsible for symptom development and disease progression.

Severity of disease differs among TBFVs. TBEV-FE is associated with the highest case fatality rate (CFR) of 20–60%, followed by TBEV-Si (CFR 7–8%) and finally TBEV-Eu (CFR 1–2%); TBEV-Si also has a tendency to cause chronic TBE [23]. OHFV is rarely known to cause neurological symptoms [6,24]. However, OHFV, KFDV, and Alkhurma virus (AHFV)—a subtype of KFDV [25]—are known to cause hemorrhagic symptoms. While significant research has been done on the role of the immune system in disease outcome, particularly in the MBFV, less is known about the viral determinants of virulence and symptom development. This review will focus on the viral proteins and mechanisms that contribute to pathogenesis and influence both neuroinvasiveness and neurovirulence properties of TBFVs. 

## 2. Effects of Viral Proteins on Virulence 

All flaviviruses share a similar organization of the virion, genomic structure, and life cycle. Virions are about 50 nm spherical enveloped particles. The envelope consists of two proteins, the envelope (E) protein, which is organized into dimers, and the smaller membrane (M) protein, which is derived from the immature prM protein. Inside the envelope is the nucleocapsid, which consists of multiple copies of the capsid (C) protein and the viral genome. The viral genome is a positive-sense, single-stranded RNA (+ssRNA) genome of about 11 kb that contains 5′ and 3′ untranslated regions (UTRs) and encodes three structural proteins (C, prM, E), and seven nonstructural (NS) proteins. The viral proteins are encoded in a single open reading frame (ORF) that is co- and post-translationally cleaved by viral and host proteases. The polyprotein is arranged 5′-C-prM-E-NS1-NS2A-NS2B-NS3-NS4A-NS4B-NS5-3′. The virus enters the cell via receptor mediated endocytosis. Following entry, the envelope fuses with the endosomal membrane, releasing the genome into the cytosol. The genome is then translated in the endoplasmic reticulum (ER), and viral particles are assembled in the ER lumen. Immature particles are then transported through the secretory pathway, where prM is cleaved to M by the host protease furin, and the mature particle is released from the cell. Genetic comparisons of strains with varying degrees of pathogenicity have revealed differences in the polyprotein, suggesting a role in virulence. The major proteins implicated in virulence are discussed below.

### 2.1. Envelope (E) Protein

The viral E protein forms the outer surface of the virion and mediates receptor binding and membrane fusion. It is also one of the primary antigenic structures of the virus. Unlike many other enveloped viruses, the E protein is oriented parallel to the viral membrane, rather than perpendicular, in head-to-tail homodimers on the mature virion surface. Following receptor-mediated endocytosis, exposure to low pH in the endosome triggers an irreversible conformational rearrangement from dimers to trimers. This rearrangement facilitates fusion with the endosomal membrane and release of the viral genome into the cytosol. 

The protein is comprised of three domains—I, II, and III—and the stem anchor region. Domain I contains an N-linked glycosylation site, domain II is involved in membrane fusion and dimerization, and domain III, an immunoglobulin-like module, is recognized as the receptor binding domain. Antigenic epitopes have been mapped to all three domains; however, cross reactive epitopes have only mapped to domain II [26]. The stem anchor region is important for protein anchoring and interactions with prM. E is a major determinant of cell tropism and virulence due to its role in viral entry and immune activation, Cell passaging, neutralizing antibody escape mutants, and reverse genetics have been used extensively to study the role of E in TBFV pathogenicity. 

Although host cell entry receptors for flaviviruses, particularly TBFVs, remain ill-defined, the glycosaminoglycan (GAG) heparin sulfate (HS) has been identified as a low affinity binding molecule [27] and is expressed on many cell types. In vitro, virus passage quickly yields virus with mutations increasing the net positive charge of E, which appears to be a mechanism of adaptation for specific cell line growth. These mutations increase viral interaction with negatively charged GAGs allowing for more efficient infection. While GAG-binding variants may have a replicative advantage in cell lines, many of these have been shown to be attenuated for neuroinvasiveness in vivo [13,28,29,30,31]. Interaction of viral proteins with GAGs may serve to concentrate virus on the cell surface and thus facilitate interaction with high affinity receptors [13]—such as D-SIGN, TIM, and TAM—which were recently implicated in MBFV entry [32] but have yet to be studied in TBFVs. However, this mechanism appears to result in limited viral spread from infected tissues in vivo*,* enhancing viral clearance and decreasing the virus’ ability to cause viremia and invade the CNS [28,33].

**Figure 1:**
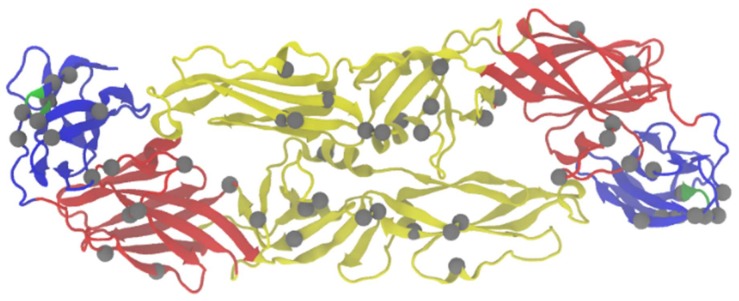
Residues implicated in virulence in the E protein. The homodimer of TBEV protein E is shown. Domain I is shown in red, domain II in yellow, domain III in blue, FG loop of domain III is noted in green. Residues implicated in virulence (summarized in Table 1) are indicated in grey. Stem anchor not shown (Residues 401–496).

Mutations increasing GAG-binding affinity have been located in all three domains on the outer surface of the protein (summarized in Figure 1 and Table 1) and usually occur near existing positive clusters [28]. However, mutations in domain II dominate, as it is an extended structure that forms a large portion of the surface of the protein. One attenuating mutation in domain II of TBEV, E122G, has arisen several times in various laboratory settings, including cell passage, neutralizing antibody escape mutants, and infectious cDNA recovery, as well as being identified in low virulence natural isolates [28,29,30,31,34]. In one study that evaluated the mutation in greater detail, the researchers found that the attenuated TBEV E122G mutant displayed a more pronounced increase in binding affinity to BHK-21 cells than did an attenuated E201K mutant compared to the wild type virus [28]. Thus, this mutation appears to be particularly advantageous for increased GAG binding, which may explain its relative prominence compared to other mutations that increase positive charge. GAG-binding variants also typically lack hemagglutinating (HA) activity. HA activity is thought to be mediated by fusion of the E protein [29]. Decreased or absent HA activity was observed to be directly correlated to GAG-binding variants carrying a mutation in the fusion domain (domain II), while increased positive charge of the E protein was not solely enough to confer this phenotype [29,30]. Increased GAG-binding affinity, particularly through mutations in domain II, appears to be a major mechanism of attenuation of neuroinvasiveness in the E protein.

In addition to the attenuating effect, GAG binding appears to facilitate host range adaptation in vivo. GAG-binding variants have been shown to exist in the natural TBEV population and these, as well as in vitro tick adapted strains, typically display HA deficiencies and small plaque phenotypes in mammalian cell lines and reduced neuroinvasiveness in mice [30,34,52]. HA deficiency, while associated with attenuation in mice, was found to directly correlate with virus titers in feeding ticks and to increase tick-to-tick transmission during cofeeding [29]. Additionally, the authors found a more hydrophobic pattern of amino acids in the E protein of *Ixodes ricinus* adapted TBEV-Eu strains compared to *I. persulcatus* vectored TBEV-Si and TBEV-FE strains. The authors proposed then that HA deficiency may be related to adaptation to new tick hosts [29]. Interestingly, this pattern also correlates with the overall reduced virulence of TBEV-Eu. Thus, variations that result in decreased virulence in humans are likely advantageous to adaptation to the tick host. 

Surface residues on the lateral side of domain III, the putative receptor binding domain, have also been identified as major determinants of virulence [43,44,45,48,49]. At least some of these mutations appear to disrupt the functional integrity of the domain. For example, Holzmann et al. suggested that the replacement of Tyr with a His residue at position 384 in a neutralizing antibody escape mutant of TBEV-Eu strain Hypr affected a structural element important for neurovirulence [48]. Position 384 is also part of a cluster of residues in the FG loop of the domain (Figure 1) that has been implicated in virulence [28,43,48,49] and is predicted to be a host range determinant [26]. In addition, mutations at position 308 have been identified in TBEV, LIV, and LGTV [42,44,45,46]. A site-directed mutagenesis study suggested that mutations at position 308 likely affected the functional integrity of the protein by disrupting the salt bridge between residues 308 and 311, rather than a specific role in receptor binding interactions [13]. Interestingly, Jiang et al. noted that mutations at 308 and 310, but not 311 in LIV neutralizing antibody escape mutants showed reduced neurovirulence [45]. This observation may be related to the GAG-binding phenotype, in that mutations found at position 308 increased net positive charge by changing the negatively charged residue to a neutral residue and freeing the positively charged lysine at 311. On the other hand, the mutation at position 311 would have increased the available negative charge in the region. Thus, it is possible that, at least in this instance, the mutation at 308 contributed to a GAG-binding phenotype, influencing virulence. Gao et al. also found a mutation at position 308 in a naturally occurring LIV antibody escape mutant with reduced neurovirulence; however, this mutation did not change the charge of residue. These data suggest that 308 is significant in determining the pathogenic properties of TBFVs, although a clear mechanism is still lacking. 

Domain I contains an *N*-linked glycosylation site, which has been implicated in viral infectivity and virulence. In one study a TBEV/DENV4 chimera lacking the TBEV E glycosylation site was found to be attenuated for neurovirulence in mice [36]. Recently, Yoshii et al. demonstrated that glycosylation was not directly required for viral entry, but lack of glycosylation lowered viral infectivity and neuroinvasiveness in mice [37]. Rather, it appears that glycosylation is required to maintain proper conformation of the E protein during virion maturation. Thus, inhibition of glycosylation results in particles with reduced infectivity. Interestingly, glycosylation was not found to be important for growth in tick cells and thus represents another adaptation to and determinant of pathogenesis in the mammalian host [37].

The stem anchor region has also been implicated in both virulence and tick host range (Table 1) [33,34,50,52]. For example, mutations clustered in the predicted alpha helices of the stem anchor resulted in lowered neurovirulence in several LGTV variants unable to bind membrane receptor preparations and neutralizing antibody escape mutants [50]. In addition, in a Mediterranean *Hyalomma* (*Hyalomma marginatum marginatum)* tick-adapted TBEV strain, substitution of Ile at position 426 conferred a small plaque, attenuated phenotype, while all large plaque revertants had the original Thr residue [34]. Position 426, which is located in the highly conserved sequence between the two alpha helices, also appears to be important for host range, as it varies in TBEV strains with different tick vectors. Thr is conserved in the more virulent TBEV-Si and TBEV-FE subtypes vectored by *I. persulcatus*, while TBEV-Eu transmitted by *I. ricinus* has Ala at this position [34]. While the mechanism behind these observations is unknown, there appears to be a relationship between virulence and tick host range, in accordance with the case fatality rate of different TBEV subtypes.

Variations in the E protein, particularly those that increase GAG binding affinity, have clear implications for host range and neuroinvasiveness in mammals and represent a major virulence factor in TBFVs. While some of these studies have indicated mutations important for neurovirulence, many of them did not test neuroinvasiveness or were conducted using the attenuated LGTV with which tests of neuroinvasion are often impractical. Most mutations in the E protein have been implicated in attenuation of neuroinvasiveness, and no mutation that specifically affects the capacity of the virus to infect neurons has yet been identified. As stated previously, mutations in the receptor binding domain are important determinants of virulence; however, it is not clear that this is due to a disruption of receptor interactions or other mechanisms such as general structural integrity of the region. Understanding the mechanisms and receptors responsible for TBFV entry and neuronal tropism will be critical for developing sufficiently attenuated vaccines.

### 2.2. Nonstructural Protein 5 (NS5)

With about 900 amino acid residues, the viral NS5 protein is the largest and most conserved of the flavivirus proteins. It contains an N-terminal methyltransferase (MTase) responsible for methylating the viral RNA cap, and a C-terminal RNA-dependent RNA polymerase (RdRp) for synthesis of the viral genome. More recently, NS5 has garnered attention for its role in immune evasion. NS5 was first identified as a suppressor of type I interferon signaling (IFN-I) signaling in LGTV and has been shown to be the most potent IFN-I inhibitor of the flavivirus proteins [53,54,55,56]. The IFN antagonist function of NS5 has been mapped mainly to residues 355–735 in the RdRp, although, at least in the case of TBEV, the MTase also plays a role [56,57]. Several residues within the 355–735 region, particularly D380 and W647, are critical for IFN antagonism and may represent a novel functional site within the RdRp [57]. 

Suppression of tyrosine phosphorylated signal transducer and activator of transcription 1 (pY-STAT1) accumulation during LGTV infection suggested that IFN antagonism occurs upstream of the JAK-STAT signaling cascade, reducing transcription of IFN stimulated genes (ISGs). In agreement with this observation, it was found that LGTV and TBEV infection downregulated cellular expression of the IFN receptor subunit IFNAR1, but not IFNAR2 [58]. This effect was attributed to NS5 interaction with the host protein prolidase (PEPD), identifying a novel, nonenzymatic function of the protein. PEPD-NS5 interaction inhibited proper IFNAR1 expression, as PEPD proved to be critical for its maturation. PEPD was identified as a cellular target of NS5 via a yeast-two hybrid analysis of residues 355–735, the putative NS5 functional site of IFN antagonism. Thus, it appears that the major mechanism behind TBFV NS5 inhibition of JAK-STAT signaling occurs via PEPD mediated downregulation of IFNAR1. In support of this, a TBEV D380A mutant deficient in IFN-I antagonism and associated with reduced ability to downregulate IFNAR1 showed reduced viremia, delayed entry into the CNS, and 100% survival in mice following infection [58]. In addition, residues close to these, namely 378 and 674, were shown to be responsible for lowered neurovirulence in an infectious cDNA clone of TBEV-FE Oshima 5–10 strain, further highlighting the region’s importance in virulence [59]. The relative ability of NS5 to antagonize IFN-I signaling appears to be a critical determinant of virulence, at least for the MBFVs WNV and JEV. However, this was not observed for TBEV and LGTV [55]. Although LGTV is attenuated, its NS5 remains a potent inhibitor of JAK-STAT signaling. Kunjin (KUN), a naturally attenuated subtype of WNV, is a weak inhibitor of IFN-I. Substituting position 653 in KUN NS5 with the virulent WNV NY99 residue (KUN S653F) improved IFN-I antagonist function, as well as increasing NS5 colocalization with PEPD [55,58]. Thus, the intact interaction of LGTV NS5 with PEPD may explain the observed difference between mosquito-borne and tick-borne viruses. It is still possible, however, that attenuated TBEV strains show reduced IFN-I antagonist function compared to more virulent strains. Regardless, NS5 IFN-I antagonism is clearly critical for immune evasion and disease pathogenesis. 

In addition to PEPD binding, PSD-95/Dlg/ZO-1 (PDZ) binding has been implicated in the IFN antagonist function of TBEV NS5 and development of neurological symptoms. PDZ domains are protein–protein interaction modules that regulate diverse signal transduction systems. These domains recognize short amino acid motifs usually at the C-terminal end of the target protein, although internal motifs have also been identified [60]. With respect to flavivirus infection, PDZ binding has been associated with IFN-I antagonism, viral replication, and disease pathogenesis [20,56,61,62]. Initially, human Scribble (hScrib), a PDZ protein involved in cell polarity and neuronal function [60,63], was found to bind to TBEV NS5 via an internal PDZ binding motif (PBM) in the MTase domain and was implicated in IFN-I antagonism [56]. Previously, NS5 had been shown to coprecipitate with IFNAR2, suggesting a mechanism of direct inhibition by NS5 [53]. The authors speculate that hScrib may function to localize NS5 to the plasma membrane and facilitate interaction of NS5 with IFNAR2. NS5 was also found to interact with ZO-1 and -2 via PDZ binding, both of which interact with hScrib [61,62]. The C-terminal PBM was shown to interact with ZO-2, supporting the idea that NS5 is shuttled to the plasma membrane in association with an hScrib-ZO-2 complex. Later, hScrib was also found to be important disease pathogenesis, as it was shown that NS5 association with hScrib suppressed neurite outgrowth in PC12 cells treated with nerve-growth factor (NGF) to induce differentiation. NS5 expression inhibited Rac1 binding with hScrib, suggesting that it interfered with proper formation of the Scrib/Rac1 complex required for neuronal differentiation [20].

Several other C-terminal and internal PDZ binding partners were identified for NS5, all which play major roles in neuronal function [61,62]. While specific mechanisms remain to be elucidated, this suggests that PDZ binding of NS5 plays an important role in the development of neurological dysfunction during TBFV infection. Interestingly, Werme et al. note that LGTV contains a Phe at residues 222 and 903, resulting in suboptimal PDZ binding of both identified PBMs, and is the only virus in the TBE complex with these substitutions [56]. Although PDZ binding has been implicated in IFN-I antagonism, the ability of LGTV to inhibit JAK-STAT signaling is comparable to that of TBEV. However, LGTV was also able to associate with PEPD and downregulate IFNAR1 expression [58]. Thus, PEPD interaction may be sufficient for robust IFN antagonism, while suboptimal PDZ binding may be more important for development of neurological symptoms. PDZ binding was also important for viral replication [62], and thus reduced binding in LGTV may affect its ability to establish peripheral viremia sufficient to invade the CNS. This further supports the idea that PDZ binding is critical for disease pathogenesis, as LGTV is naturally attenuated in humans; however, this remains to be confirmed experimentally.

Multiple studies have identified other mutations in NS5 associated with reduced virulence; however, the mechanisms behind these effects are largely unknown. Many of these mutations have been identified in the RdRp domain and may disrupt polymerase activity or IFN-I antagonism. For example, D836G was identified in an infectious cDNA clone of OHFV. This substitution disrupts a salt bridge within NS5, and it was speculated that it may cause structural fluctuation and affect *de novo* initiation of RNA synthesis [64]. Additionally, comparisons of strains isolated from patients infected with TBEV identified several differences in NS5 across strains with varying disease outcomes. Interestingly, two studies identified mutations at position 692, where Ile correlated with high pathogenicity strains while Val was associated with low pathogenicity [65,66]. A third study compared strains isolated from patients with severe TBE found Ile or Thr at position 692; Thr has only been found in European human pathogenic strain Ljublijana I [67]. The authors speculate this substitution may improve interaction with host replication trans-acting factors. Mutations S634T/Y, R677K, and A724S were also associated with varying degrees of pathogenicity (substitutions associated with high pathogenicity strains are noted first, followed by the residue position and the low pathogenicity substitution), although their role in virulence has not been tested experimentally [65,66]. It should be noted that these mutations also reside within the IFN-I antagonism functional region. 

Although it is generally uncertain how TBFVs cause an encephalitic versus hemorrhagic phenotype, a study comparing TBEV and OHFV recently identified a role for NS5 in the development of neurological symptoms [21]. Infection of mice with OHFV containing the TBEV NS5 caused neurological disease in 88.9% of mice. The region responsible for this observation was further narrowed to the C-terminus of the RdRp, in which TBEV and OHFV differ by only four amino acids, 879–881 and 891. Introduction of all four TBEV residues, termed the KFK-D motif, into OHFV NS5 caused a significant increase in the development of neurological disease, similar to that seen with TBEV. Confirming the importance of these residues, substitution of the OHFV residues into TBEV reduced neurological disease. Importantly, they did not affect the morbidity, mortality, or survival curves of either virus, highlighting their role in disease phenotype rather than virulence per se. These residues were highly conserved in all TBEV strains evaluated and differed from hemorrhagic virus strains. Interestingly, LGTV also differed at these residues compared to TBEV strains. 

Thus far, it appears differences in the region responsible for IFN-I antagonism, and possibly PDZ binding, are the main determinants of virulence within NS5. Aside from the importance of IFN-I antagonism for viral replication and immune evasion on the cellular level, loss of IFN-I signaling early in infection likely has larger implications for disease progression as it inhibits proper maturation of dendritic cells (DCs) and induction of T cell proliferation [68]. DCs are early targets of flavivirus infection, and IFN-I signaling serves to induce DC maturation and thus activation of adaptive immunity. Therefore, differences in IFN-I antagonist ability would likely determine the strength of the cellular antiviral response as well as the systemic response. Further characterization of the IFN-I antagonist function of NS5 will be important for clarifying its role in determining virulence. 

### 2.3. Other Viral Proteins

Mutations associated with differing pathogenicity have been identified in both the coding and noncoding regions of the flavivirus genome through genetic analysis of natural isolates [49,65,66,67,69] and mutations derived during infectious cDNA recovery [31,51,64]. Most of these have not been studied via reverse genetics, and thus their contribution to flavivirus virulence remains unexplored. Nevertheless, it is conceivable that these mutations have a cumulative effect [51]. Substitutions found in naturally attenuated isolates are likely more informative, as they represent mutations that have developed and been maintained within the natural TBEV population, whereas it is unclear whether mutations arising during laboratory manipulation would withstand selective pressure in the natural TBFV life cycle. Two such mutations, a deletion at position 111 in the capsid protein and Ser45Phe in the NS3 protein, have been consistently correlated with reduced pathogenicity in natural TBEV isolates [65,69,70]. Both mutations affect proper cleavage of the polyprotein, as the deletion in C occurs in a cleavage signal domain and the mutation at position 45 of NS3 may interfere with efficient catalytic activity [71].

In addition to NS5, NS4B has also been implicated in IFN antagonism, although to a lesser degree [54,55]. The function of NS4B as an IFN antagonist was shown to be dependent upon a 23 amino acid signal peptide derived from the NS4A sequence (together termed 2KNS4B) and was enhanced by the addition of NS2A and NS4A. Interestingly, TBEV and LGTV NS4B showed reduced ability to antagonize IFN signaling compared to MBFVs, with TBEV NS4B being the weakest antagonist [55]. NS1 is immunogenic and was recently shown to stimulate reactive oxygen species (ROS) production and the antioxidant defense Nrf2/ARE pathway during TBEV infection in vitro [72]. Analogous to IFN antagonism, the relative ability of different strains to stimulate an immune response via NS1 is likely an important virulence factor, however this has not been studied extensively in the TBFVs.

Another recent study demonstrated a positive correlation between the intrinsic disorder, or the degree to which a protein or portion of a protein has a specific structure under physiological conditions, of M and C and the case fatality rate of different TBEV subtypes [73]. The authors hypothesize that increased disorder allows for more promiscuous binding with proteins, fatty acids, and nucleic acids, which could facilitate replication, immune evasion, or cell tropism. Further understanding of the mechanisms of virus–host protein interactions that contribute to pathogenesis is critical. 

## 3. Effects of the Untranslated Regions (UTRs) on Virulence

The 5′ and 3′UTRs contain conserved secondary structures, stem loops (SL), and RNA elements causing cyclization of the genome in a panhandle structure important for virus translation, replication and encapsidation [74]. The length of TBFV 5′UTR is about 130 nucleotides (nt). In contrast, the 3′UTR ranges between 350 and 700 nt and exhibits great heterogeneity in both length and sequence even within the same flavivirus subtypes. Both the 5′ and 3′UTR can be divided into a highly conserved region and a variable region (5′VR and 3′VR, respectively) [75,76,77] .

### 3.1. 5′UTR

The 5′UTR region contains several conserved SLs and elements important for long distal RNA-RNA cyclization at the initial phase of replication [76,78]. Although sequence and length differences exist between flaviviruses, some SL structures remain conserved, such as the large SLA located at the end terminus of the 5′UTR, which is folded into a Y shaped structure and is essential for NS5 recruitment [78]. SLB is situated directly downstream of SLA within the 5′UTR and contains the important long-range CS motif (CS-A) [78]. The upstream of AUG region (5′UAR) elements at the genomic termini are critical for viral RNA replication. Elements downstream of AUG region (5′DAR), situated in the N-terminal region of the ORF, harbor a hairpin structure (cHP) required for viral RNA replication and a suggested function in translation by stabilizing the initiation of ribosomal attachment to the AUG site [78]. Furthermore, the cHP might also play an additional role in stabilizing the panhandle structure of the genome and recruiting replication factors [79]. Clearly, disruptions in the 5′UTR that interfere with proper genome cyclization and viral replication have implications for virulence and lethality of the virus. However, major differences in the 5′UTR that still result in naturally viable strains have not been identified, and thus the 5′UTR likely does not have a major role in determining virulence. 

### 3.2. 3′UTR

**Figure 2:**
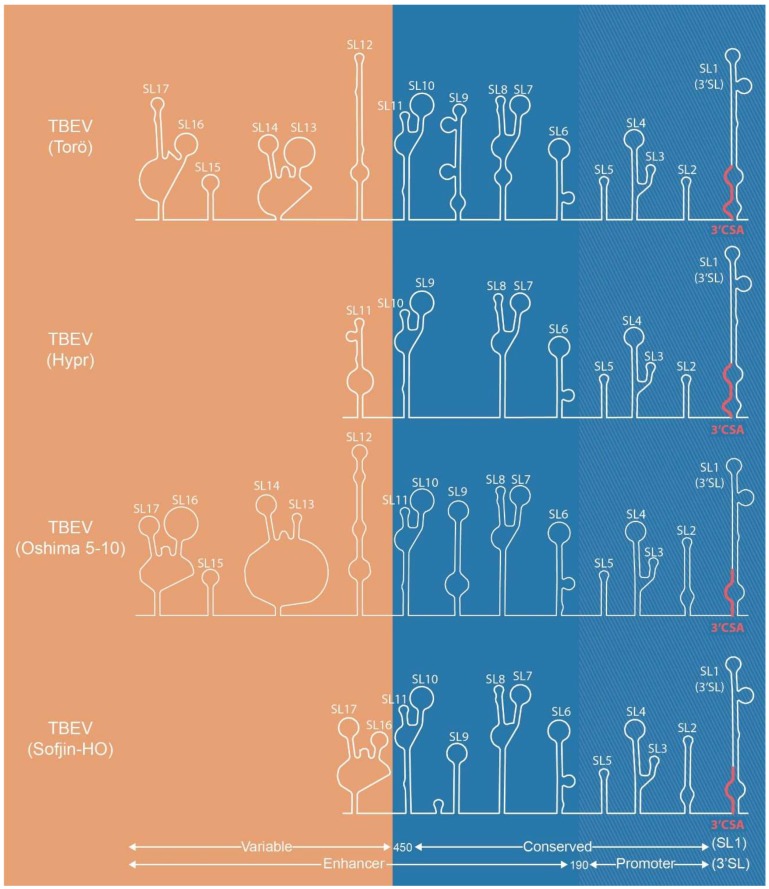
Predicted secondary structure of the 3′UTR of TBEV-Eu strains Torö (accession no. DQ401140) and Hypr (accession no. U39292), and TBEV-FE strains Oshima 5-10 (accession no. AB062063) and Sofjin-HO (accession no. AB062064). Secondary structures were predicted using Mfold (http://unafold.rna.albany.edu/?q=mfold/RNA-Folding-Form). The 450 nt conserved region of the UTR (shown in blue) is comprised of the enhancer element and the terminal 190 nt promotor element, with the promoter element, including the 3′SL, being essential for replication. The nucleotides between the coding region and the terminal end of the UTR are referred to as the variable region (shown in orange). Variation in the length of the UTR among strains is due to deletions in the variable regions and correlates to virulence.

The 3′UTR is longer and more variable than the 5′UTR and, in addition to its critical role in viral replication, contains structures relevant to immune evasion and viral pathogenicity. The terminal 190 nucleotides (nt) of the 450 nt conserved region of the 3′UTR contain essential elements in the viral lifecycle: the 3′-terminal SL structure (3′SL), which shows a high degree of conservation among all flaviviruses, the CS motifs complementary to CSA on the 5′UTR end of the genome and a short hairpin adjacent of the 3′SL (SL2) (Figure 2) [80]. The 190 nt 3′UTR terminal end, referred to as the promoter element, exhibits additional conserved SLs (SL2-SL5) shared by all TBFVs [75,81]. Numerous studies using either a subgenomic replicon system or a cDNA infectious clone of both MBFVs and TBFVs to introduce progressive SL deletions in the promoter element toward the 3′ terminus have shown a significant impact on RNA synthesis as the deletions progress inward, while deletions or mutations that disrupt SL2 and 3′SL abolish virus replication [82,83,84,85,86,87]. The remaining part of the conserved region (190–450 nt) contains additional SLs important for virus replication and is referred to as the enhancer element [75,88,89]. Progressive deletions inward of the enhancer element attenuate viral replication and reduce RNA synthesis. 

The 3′VR starts immediately downstream of the stop codon of the viral ORF and upstream of the conserved region and is extremely heterogeneous in both length and sequence in natural isolates [90,91,92]. The role of 3′VR is more elusive than other regions of the UTRs and is generally thought of as an enhancer element. Recently, it has also garnered interest as a virulence factor. An early observation noted that passage in cell culture often resulted in deletions within the VR, which is proposed to be related to host adaptation [66,67,88]. Interestingly, this observation correlates with pathogenicity in mammals. For instance, the Hypr strain lacks most of its 3′VR and is considered the most virulent strain within TBEV-Eu, and TBEV strains isolated from brain tissue of patients with severe cases of encephalitis consistently display deletions in the 3′VR [67,69,88,93]. The mortality rate across the three subtypes—TBEV-FE, TBEV-Si, and TBEV-Eu—correlates with the length of the 3′VR among the subtypes as well, where members of TBEV-Eu contain the longest 3′VRs overall compared to those of TBEV-FE strains [23,25,75,94].

Initially, the VR was not thought to affect flavivirus biology, as the entire VR of the TBEV-Eu Neudoerfl strain was deleted without any significant effect on virus pathogenicity [88]. Recent work by Sakai and colleagues, however, demonstrated that the 3′VR is a critical determinant of virulence in infected five-week-old female C57BL/6 mice [70,95]. The authors used infectious cDNA clones of the highly virulent TBEV-FE strain Sofjin-HO (Sofjin-IC) and low virulence TBEV-FE strain Oshima 5-10 (Oshima-IC), which differ by 44 amino acids across the viral genome as well as a deletion of 207 nt in the 3′VR of Sofjin-HO, to identify regions responsible for the difference in virulence between the two strains. An Oshima-IC chimera containing sequences from the coding region of Sofjin-IC resulted in a limited increase in viral pathogenicity in C57BL/6 mice. Interestingly, the Oshima 5-10 chimera containing the Sofjin-IC 3′VR killed 100% of the mice, with a virulence profile similar to that of the parental Sofjin-IC. Furthermore, the chimeric Oshima-IC containing the virulent VR showed histopathological changes in the brain tissue of infected mice similar to Sofjin-IC [70]. The authors suggest the discrepancy between this and the previous work with Neudoerfl may be because Neudoerfl is already highly virulent in the mouse model (LD_50_ < 10), resulting in an undetectable increase in virulence following deletion of the VR. 

Secondary structure predictions showed three SL structures in the Oshima VR, designated SL3, SL4, and SL5 by Sakai and colleagues (corresponding to SL15, 14, 13, and 12, respectively, in Figure 2), within the deletion region of the Sofjin VR. To continue investigating the detailed function of the variable region, the researchers constructed infectious cDNA mutants with progressive deletions removing these structures. In C57BL/6 mice, the deletion mutants had virulence similar to that of Sofin-IC [95]. Disruption of SL3 and SL4 was proposed to be responsible for this effect, as deletion of SL5 also affected the predicted structure of these SLs as well. Sakai et al. suggested the observed increase in virulence could be due to the role of the 3′VR in RNA-protein interaction with host factors [95]. Indeed, several proteins involved in neuronal function were found to bind to the full length Oshima VR, as well as the mutant missing SL3/4 or SL5, but not a mutant missing SL3-5 [96]. One of these proteins, FMRP, influenced viral replication as well. FMRP knockdown reduced viral replication, while deletion of SL3-5 slightly recovered viral titer. This study indicates that FMRP plays a role in TBEV replication in vitro, although how the loss of SL3-5 compensates for FMRP involvement is unclear. Interestingly, FMRP also binds to the 5′UTR. 

Prion-like T-cell-restricted intracellular antigen 1 (TIA-1) and TIA-1 related protein (TIAR) were also found to bind the 3′UTR of Neudoerfl and exhibited a suppressive effect on viral replication [97]. Although the UTR region responsible for this interaction was not further specified, this supports the notion that the 3′UTR structures may also interact with host factors responsible for protective immune responses. Thus, deletion of certain structures in the 3′UTR could serve as an immune evasion strategy as well. 

Sakai and colleagues also demonstrated the role of a naturally introduced poly(A) tract as a virulence factor. A 35 nt stretch of A was introduced into the full-length 3′VR of Oshima-IC, where Sofjin-IC contains a deletion. The recombinant Oshima-IC-poly(A) mutant resulted in the same level of mortality as seen with Sofjin-IC infection [95]. The role of the poly(A) tract as a virulence factor was further supported using two infectious cDNA clones of the TBEV-Eu strain Torö-2003, one with the shorter wildtype poly(A) tract and one with an extended tract [98]. Torö-38A, which contained the extended poly(A) tract, was significantly more neuroinvasive and neurovirulent than the short tract Torö-6A clone. The long poly(A) tract was also genomically much more unstable than the shorter tract, resulting in a more diverse quasispecies pool following infection. The authors speculate the genomic instability of the poly(A) tract and resultant diversity of quasispecies facilitates rapid host adaptation and fitness selection, which may be related to the observed correlation with pathogenicity [98]. In conjunction, a TBEV strain isolated from a tick feeding on a human was heterogeneous in its poly(A) tract compared to an absent poly(A) tract in a strain isolated from field collected ticks [99]. The authors suggest the mixed environment within the feeding tick led to rearrangements in the quasispecies pool to select for best-fit variants. 

### 3.3. sfRNA

In addition to its role in genome cyclization and translation, the 3′UTR is important in the synthesis of subgenomic flavivirus RNA (sfRNA). Following decapping of +ssRNA or upstream cleavage in the genomic RNA, host 5′–3′ exoribonuclease (XRN1) generates sfRNA (full length, sfRNA1) via stalling on SL structures during viral RNA degredation [77,100,101]. sfRNA was first observed for MBFVs: WNV, JEV, and Murray Valley encephalitis virus (MVEV), and was investigated more extensively for its role in WNV pathogenesis [77,102]. Several wild type MBFVs (YFV and DENV) isolates from infected mosquito and mammalian cell culture or mice brain also produce multiple endogenous truncated sfRNA, suggesting a ‘slipping’ effect of the stalling XRN1 and involvement of downstream SL structures resulting in shorter sfRNA (sfRNA2, sfRNA3, and sfRNA4). The biological role of sfRNA and the mechanism by which it influences viral pathogenicity is as yet unknown. One hypothesis that has gained traction is a role for sfRNA modulation of host antiviral responses by antagonizing or inactivating host factors in the IFN-mediated innate immune response [77]. Another speculation is that sfRNA functions as a microRNA (miRNA) decoy to inhibit host mRNA silencing pathways [103]. While TBFV production of sfRNA in vertebrate and invertebrate cells has been demonstrated [104,105], its role in pathogenesis has not been evaluated in vivo. Recognition of sfRNA is relatively new, and most work to date has focused on MBFVs. Thus, additional research into the role of sfRNA in TBFV infection is warranted and represents an important avenue of research for understanding TBFV pathogenesis.

## 4. Conclusions

Although TBFVs are emerging and reemerging pathogens with a significant disease burden, they receive much less attention than their MBFV counterparts. Determining virulence factors and uncovering the mechanisms behind TBFV pathogenesis will be critical for the development of vaccines and therapeutics. The ability to influence virulence, in particular neurovirulence, without interfering with immune activation is of particular importance in the development of safe and effective vaccines against TBFVs. To this end, residues affecting neurovirulence remain to be identified. As discussed previously, no mutations in the E protein have been identified that reduce neurovirulence specifically, without affecting peripheral infectivity and neuroinvasiveness as well. Identifying high affinity or cell specific entry receptors will also be of importance. In addition, the 3′VR and sfRNA production may play a major role in TBFV pathogenesis but remain to be explored in greater depth. Uncovering host–virus protein interactions responsible for immune activation and the development of neurological symptoms and damage will be critical for developing therapeutics, as treatment for TBFV infection is currently supportive. 

## Figures and Tables

**Table 1:** Mutations affecting neuroinvasiveness and neurovirulence in the E protein.

Virus	Domain	Substitution	Reference
LGTV	I	E149G	[35]
TBEV	I	N154L	[36]
TBEV	I	N154Q	[37]
TBEV	I	S158R	[28]
TBEV	I	G159R	[28]
TBEV	I	K171E	[38]
TBEV	I	D181Y	[39]
LGTV	I	G285S	[40]
TBEV	II	D67G	[29,30,38]
TBEV	II	T68A	[30]
TBEV	II	E84K	[28,41]
LGTV	II	F119V	[40,42]
TBEV	II	E122G	[28,29,30,31,34]
TBEV	II	A123K	[28,39]
LGTV	II	H130Y	[43]
TBEV	II	E201K	[28]
TBEV	II	D203G	[28,33]
LGTV	II	S267L	[43]
TBEV	II	D277A	[29]
LGTV	III	D308A	[42]
LIV	III	D308N	[44,45]
LIV	III	S310P	[45]
TBEV	III	T310K	[28,46]
TBEV	III	D308K/K311E	[46]
LGTV	III	K315E	[43,47]
LGTV	III	F333S	[40]
TBEV	III	G368R	[39]
TBEV	III	Y384H	[48]
LGTV	III	N389D	[40,42,43]
TBEV	III	H390Y	[49]
LGTV	Stem Anchor	L416A	[50]
TBEV	Stem Anchor	T426I	[34]
LGTV	Stem Anchor	H438Y	[42,50]
LGTV	Stem Anchor	V440A	[50]
LGTV	Stem Anchor	N473K	[50]
TBEV	Stem Anchor	D483E	[33]
TBEV	Stem Anchor	H496R	[51]
